# German Physicians and Medical Students Do Not Represent the Population They Serve

**DOI:** 10.3390/healthcare11121662

**Published:** 2023-06-06

**Authors:** Oana R. Groene, Thorben Huelmann, Wolfgang Hampe, Pedram Emami

**Affiliations:** 1 Institute of Biochemistry and Molecular Cell Biology, Center for Experimental Medicine, University Medical Center Hamburg-Eppendorf, 20246 Hamburg, Germany; 2 Hamburg Chamber of Physicians, 22083 Hamburg, Germany

**Keywords:** diversity, equity, physicians, medical students, widening participation

## Abstract

Medical professionals who represent the communities they serve are in a better position to understand patients‘ social circumstances and communicate in a more patient-centered way. International studies show limited diversity and underrepresentation of certain social groups in the population of physicians and medical students. We designed an observational study to investigate the cultural and socio-economic diversity of physicians and medical applicants in comparison to the general population in Germany. We invited 15,195 physicians in Hamburg and 11,287 medical applicants in Germany to participate in an online survey between June and August 2022. The lower three quintiles of objective socio-economic background (SEB) were vastly underrepresented in all subsamples of the study and in particular amongst applicants and students admitted in Hamburg: 57.9% of physicians and 73.8% of medical students in Hamburg originate from the top quintile of SEB. The Turkish and Polish communities were particularly underrepresented in the group of physicians from Hamburg and medical applicants and students in Germany (*p* = 0.02; *p* < 0.001). In line with existing evidence, the vast majority of physicians and medical students come from the most affluent households when entering medical school. Widening participation strategies are needed to facilitate fairer access to the study of medicine in Germany.

## 1. Introduction

It is much easier for a medical professional to build a relationship with a patient if both come from the same cultural and socio-economic background. Patients report greater satisfaction with treatment and improved communication experiences [1]. Research on health inequalities also indicates reduced access to healthcare and discriminatory experiences of cultural minorities: when treating patients from a different cultural and lower socio-economic background, providers demonstrate implicit bias and prejudice most likely due to a lack of cultural competence or unconscious bias [2], which can have implications on health outcomes [3,4]. Patients often complain in such situations about a lack of empathy and a failure to understand the doctor’s communication style, which is also reflected in lower adherence and inadequate use of preventive and health services [5,6]. This compelling evidence suggests that it can be beneficial for healthcare quality if medical professionals represent the communities they serve, particularly because of their ability to understand the social circumstances of health and illness and communicate in a patient-centered way.

Continuous societal changes and the increasing diversity of the population require the diversification of the medical profession, whose benefits are often emphasised as a strategy to reduce health disparities and improve access to high-quality care for minority or underserved populations [7]. Diversifying the healthcare force also increases the chances of a doctor-patient cultural and/or socio-economic match as studies show that physicians from minority groups are more likely to practice in underserved communities [8,9]. One way this can be attained is through widening participation strategies aimed at recruiting students from disadvantaged or minority backgrounds. Such strategies include contextualizing admission criteria for health-related undergraduate programs that may be otherwise challenging for disadvantaged students [10].

Studies in the USA report on a lack of ethnical diversity, with black and Hispanic people being particularly underrepresented in the group of physicians [11,12,13,14,15,16]. Recent research suggests that inequalities in access to the profession have not changed in the past decade: concerning students‘ socio-economic background, an overrepresentation of high-income households has remained stable [17]. This situation is also known in the UK, where 80% of medical school applicants come from a low number of schools and colleges, more likely to be expensive or private [18]. Moreover, up to 34.5% of UK applicants live in the most affluent tenth of postcodes and have better chances of being admitted into medical schools than applicants from less wealthy backgrounds [19]. This evidence suggests that current education systems may be perpetuating issues around inequalities between different population groups. This deserves attention, as it is a societal responsibility. In Germany, 80% of children coming from academic families go into higher education compared to only 27% of children from non-academic families [20]. About 70% of medical students have at least one parent with a university degree [21,22] although in the general population, this level of education is achieved by only 20% of 40- to 60-year-old people. While the population is becoming increasingly diverse [23], the population of medical students remains homogeneous. Thus, medical students do not seem to represent the population whose health problems, beliefs, and needs they are supposed to understand. Whereas in other countries, medical education stakeholders try to address the issue of the unequal representation of population groups in medicine [10,24], the discourse around unequal access to the study of medicine is missing in Germany. At the same time, German studies on medical students‘ and physicians’ socio-economic and cultural background are lacking. In this study, therefore, we explore the diversity of physicians in the city of Hamburg and German medical applicants and students from the socio-economic and cultural point of view.

## 2. Materials and Methods

### 2.1. Study Design, Participants, and Data Collection

This is an observational study using an online questionnaire to explore the socio-economic and cultural characteristics of participants from two different groups: physicians in Hamburg and medical applicants in Germany. All 15,195 physicians registered with an email address at the Hamburg Chamber of Physicians (HCP) were invited to take part in the online SEKHHA survey (socio-economic and cultural background of physicians in Hamburg). In the stav survey, we invited 11,287 medical applicants and students who had agreed to participate in research projects upon taking admission tests, of which 2013 (17.8%) were included.

In both cases, the invitations were sent by email and the data were collected between June and August 2022. Inclusion criteria consisted of being a member of HCP (for physicians), applying for medicine or dentistry (for applicants), and participants answering a sufficient number of questions to determine either their cultural or socio-economic background.

### 2.2. Instruments

We developed an online questionnaire that contained items related to participants’ gender, type of secondary school attended, final secondary school grades (referred to as high school Grade Point Average-GPA), and native language. We used previously validated instruments from the national KiGGs study (German health interview and examination survey for children and adolescents) [25] for the measurement of the main outcome variables. The objective socio-economic background (SEB) was calculated taking into account income, parents’ education, and occupation at the time of application for medical school. For the computation of the SEB, point values were calculated for each individual dimension. With regard to education and occupational status, the higher score of the parents was taken into account. For each dimension, a minimum of one and a maximum of seven points were assigned. The individual dimensions were thus included equally in the SEB score. Quintiles were estimated based on the distribution of the score in the general population. For the subjective SEB participants reported on their perceived socio-economic status. As for the description of migratory background, we collected data on participants’ citizenship and participants’ and their parents’ places of birth [25]. Having a migratory background is defined as participants or one of their parents being born in a country other than Germany. We used migratory background as a proxy for cultural background.

In order to improve the comprehensiveness and readability of items, the questionnaire was pretested with a group of 10 physicians.

### 2.3. Data Analysis

Assuming small effect sizes, we estimated in a power analysis that a minimum sample size of 600 participants in each survey would be sufficient to detect subgroup differences. Descriptive statistics were used to illustrate the socio-economic and cultural characteristics of participants. We carried out binomial tests to investigate significant differences in the proportion of migratory background distribution when comparing the physicians in Hamburg and the medical applicants and students in Hamburg and Germany to the general population. Whenever the availability of census data allowed it, we age-matched the subgroups of medical applicants and students to the corresponding general population. We focused in our analysis on the four most frequent cultural groups in Hamburg and Germany. To address issues with multiple testing we implemented Bonferroni-Holm corrections [26]. We used predictive mean matching [27] for missing values, particularly for the income variable which had between 35 and 39% missing data. A logistic regression was calculated to explore if the probability of a medical applicant being admitted into a German medical school can be determined by socio-economic and migratory background by adjusting for high school GPA and gender [28]. Although not the main purpose of the study, the logistic regression is relevant to the field of study, particularly with regard to practical implications and recommendations regarding widening participation policies. The four independent variables considered as predictors generally constitute the main criteria based on which international universities recruit widening participation students. In order to more easily interpret odds ratios for numeric independent variables, we calculated the relative likelihood as follows: OR^(Maximum reported value-Minimum reported value) [24]. All calculations were computed with SPSS 27, R 4.1.2. [29], and the R package mice [30].

## 3. Results

In total, 1812 physicians and 3336 medical applicants and students took part in the SEKHHA and stav surveys, respectively. Excluded from the study were physicians who were not HCP members (n = 2), physicians (n = 353) and medical applicants (n = 1188) who answered an insufficient number of survey questions, and candidates who applied for veterinary medicine or pharmacy (n = 135). Our final sample included 1457 physicians in Hamburg (9.6%) and 2013 medical applicants in Germany (17.8%).

The majority of Hamburg physicians were female, an even higher proportion of women apply for and are admitted into Hamburg medical school (Table 1). Across all study subsamples, participants were in their majority German citizens, German native speakers, and graduated from grammar school (“Gymnasium” in German). The high school GPA of applicants and students was on average better than that of Hamburg physicians. The proportion of Hamburg medical applicants (20.1%) who had at least one parent working as a physician was similar to that of Hamburg physicians (21.4%).

**Figure 1:**
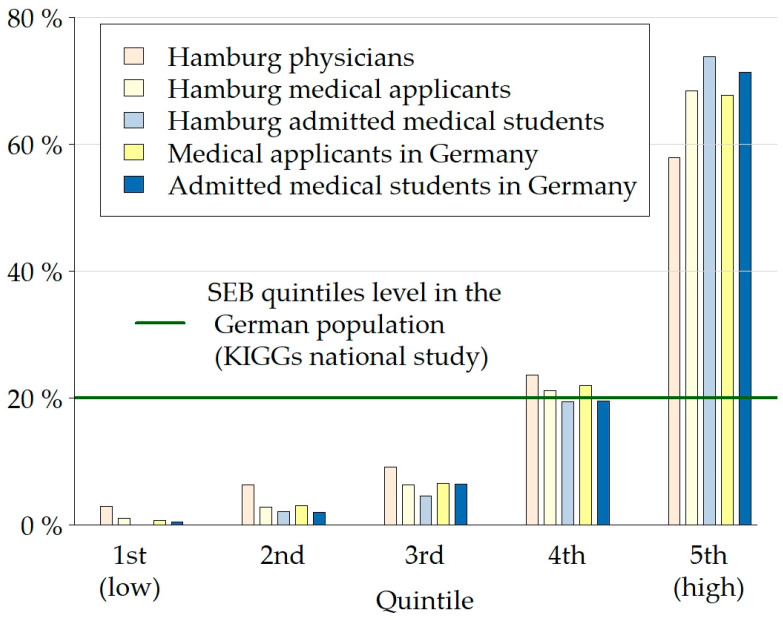
Distribution of quintiles of objective socio-economic background scores (SEB) for the subgroups of the study.

Compared to Hamburg physicians, an even higher proportion of medical applicants in Germany relied on their parents’ support to finance their studies. The average objective and subjective SEB at the entrance into medical school was similar across all subgroups in the study, with Hamburg physicians having a slightly lower mean objective SEB compared to medical applicants and students (Hedges g = 0.4; *p* < 0.001). The objective and subjective SEB correlated at ρ = 0.6 (*p* < 0.001). Figure 1 illustrates that the lowest three quintiles of objective SEB are vastly underrepresented in all subsamples of the study and in particular amongst applicants and students admitted in Hamburg.

**Figure 2:**
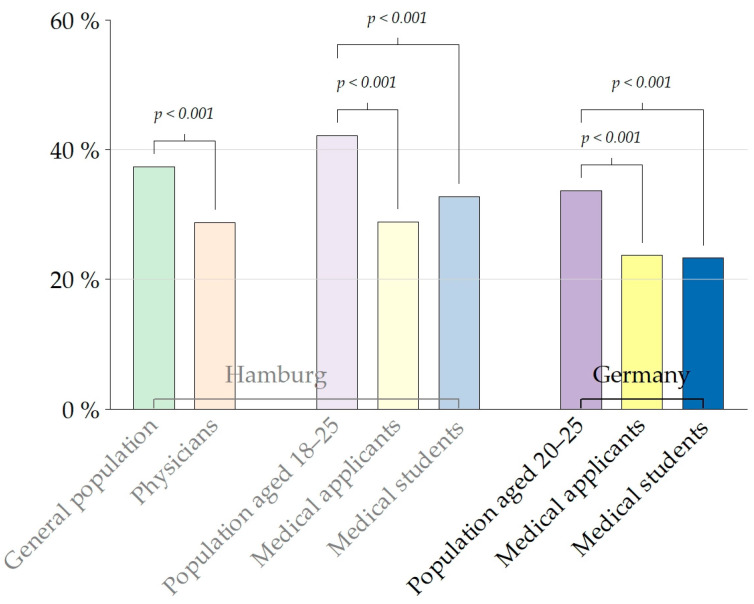
Proportion of study participants with migratory background compared to census data.

**Figure 3:**
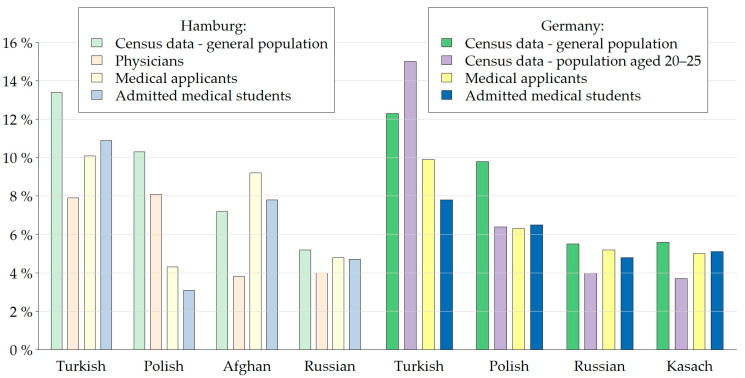
Main cultural groups in our study compared to census data on people with migratory background in Hamburg and Germany.

Physicians, applicants, and students with migratory background are underrepresented compared to the general population and these differences are statistically significant (Figure 2, Table 2). People with Turkish roots are underrepresented amongst Hamburg physicians (*p*-value < 0.001) and German medical applicants (*p*-value = 0.002) and students (*p*-value = 0.001) (Figure 3). The proportion of applicants with Polish roots who apply for medicine in Hamburg is also lower than the one in the general Hamburg population with migratory background (*p*-value = 0.02). The Afghan community is underrepresented in the population of physicians in Hamburg (*p*-value = 0.006). As shown in Table 2, compared to the general population in Germany aged 20 to 25, a much lower proportion of young people with Syrian roots in Germany apply for and are admitted into medical schools (*p*-value = 0.001 for both subgroups).

Overrepresented in the SEKHHA study were Hamburg physicians with Iranian (8.3% compared to 3.8% in the Hamburg general population with migratory background) and Austrian roots (5.7% compared to 1.1%). In the subgroup of applicants in Germany, we similarly found an overrepresentation of people with Iranian (5.4%) and Austrian (3.6%) roots; these proportions remained almost the same after admission into medical school: Iranian roots (5.4%), Austrian roots (2.8%).

High school GPA, objective SEB, and gender contributed significantly to the probability of being admitted into medical school according to the logistic regression model which correctly classified 64.8% of cases overall (Table 3). For one unit change in the SEB, there is an Exp (B) change of 1.07 in the outcome, which means that students with the highest SEB score are 3.1 times more likely (1.07^(21–4.4)) to be admitted into medical school than students with the lowest SEB score, after adjusting for high school GPA and gender. The effect of the migratory background was not statistically significant in our sample. We additionally conducted a sensitivity analysis to test how the probability of being admitted into medical school can be predicted by migratory status, gender, and SEB alone; in this regression model, the Exp(B) for SEB was 1.085 (*p*-value < 0.001) while migratory background and gender became statistically insignificant.

## 4. Discussion

In this study, we present evidence on the diversity of physicians, medical students, and applicants, which has not been available so far for Germany.

### 4.1. Socio-Economic Diversity

The great majority of medical applicants, students, and physicians came from the top two quintiles of the SEB score. The overrepresentation of the highest quintile was particularly manifest amongst medical students indicating that inequalities in accessing medical studies in Germany have at least maintained if not worsened, a finding consistent with existing international evidence [31]. Moreover, as similarly found in a Swedish study [32], medicine appears to stay in the family, as one in five study participants had at least one parent that was a doctor.

As in many other countries [33], high school GPA is an important selection criterion for medical students in Germany and can predict study success [34]. However, young people with low socio-economic status are strongly underrepresented among school leavers with good grades that are needed for admission into medical school. The link between social class and education is particularly pronounced in Germany where PISA assessment results depend on individual socio-economic status to a greater extent than in the other OECD countries on average [35]. Therefore, the underrepresentation of the lower SEB among medical applicants could also result from self-selection, as pupils from underrepresented groups may not even consider studying medicine or may not have the confidence to do so.

### 4.2. Cultural Diversity

In line with international studies, our research indicates that Hamburg physicians and medical applicants in Germany form a culturally homogenous group [14,19]. The underrepresentation of participants with migratory background in German higher education has been previously reported on in education reports: persons of the same age with a migratory background are less likely to have a university degree and more likely to have no vocational qualification [36]. One explanation for this type of underrepresentation is that migratory background is closely linked to socio-economic status. This fits our observation that the underrepresentation is especially dominant for cultural groups with a low SEB in Germany [17,37]. It is for example well known that people with Iranian roots living in Germany are amongst the most educated and generally have a very good income [38]. Our logistic regression model similarly suggests a higher effect of socio-economic status compared to migratory background.

### 4.3. Practical and Political Implications

The underrepresentation of certain groups in the medical workforce has two main implications: questionable fairness in the selection of medical applicants and worse quality of healthcare.

Regarding fairness in student selection, each applicant should have a fair chance to enter medical school, regardless of socio-economic or cultural background. German politics can benefit from experiences in other countries where strategies to increase the chances of students from disadvantaged backgrounds to study medicine have been implemented as briefly mentioned in the introduction under the name of “Widening Access” or “Widening Participation” [10,39,40]. In Canada, it is ensured that members of underrepresented groups are involved in the development and assessment of admission procedures [41,42]. In the Netherlands, the selection by lottery will be reintroduced after years of using admission tests [43]. In the USA, quotas and bonuses for minority groups are used [44,45]. This “affirmative action” approach is controversial and subject to legal action. In the UK, adjusted criteria for applicants with low SEB are implemented for contextualized admissions; in one medical school, every applicant’s high school GPA is considered in comparison to the average school attainment, which proved to have a significant effect on widening participation [46]. Moreover, recruitment campaigns are organized in disadvantaged areas to encourage pupils from underrepresented groups to inform, empower, and raise the aspirations of these groups to apply to medical school. A combination of mentoring programs and summer schools has proved to be successful [10].

Student selection in Germany is highly regulated by law. A sentence of the constitutional court in 2017 has led to a reform of selection rules, which medical schools have to apply. The question remains whether there is a political will to create a legal framework that could accommodate the implementation of widening participation approaches in Germany.

Following the argument of healthcare quality, the need for a diverse healthcare has been emphasized in German health policy documents which underline the importance of designing “diversity sensitive healthcare” [47]. A diverse healthcare force has the potential to improve the cultural competencies of healthcare staff and the health outcomes of patients. Although there is no guarantee that physicians from a minority group will serve in their own community, international studies show that this can be achieved [8,9]. Human resources for health migration is a phenomenon that partially led to increasing diversity; however, decision-makers need to consider the disastrous consequences for the health personnel’s country of origin such as insufficient healthcare delivery and increased mortality [48,49]. The current evidence suggests that strategies to obtain a more diverse healthcare force should rather focus on the recruitment of local talent.

Medical education alone cannot solve the issue of underrepresentation of population groups in higher education and widening participation is surely an issue that needs to be addressed by all sectors of society, particularly the school system. Widening participation interventions need to be initiated at an early educational stage so that each child can optimally develop their abilities. However, in order for their graduates to be able to address the diverse preferences, communication needs, and demands of all patients, medical schools must consider criteria other than merit and academic achievement.

### 4.4. Limitations

With participation rates below 20%, we cannot state with confidence that our findings are generalizable. Our sample size calculation indicated that the sample is sufficient to generate solid findings; however, some comparisons for certain cultural groups were not meaningful because of the very small number of cases in each subgroup. Future research should be conducted at the national level to gain a better insight into the diversity of the medical workforce and studentship across Germany.

We have not performed any parity adjustment for income in the case of people whose parents may have been employed in a different country; therefore, we may have underestimated some participants’ socio-economic background scores. For the income variable, we had a high number of missing data (between 35 and 39%); given the good correlation between the objective and subjective socio-economic background, researchers should consider in the future the use of subjective measures. Alternatively, the school social index (currently used in Hamburg, Germany) could also constitute a good measure for socio-economic disadvantage.

Our study could not establish a direct causal link between the socio-economic status and access to the medical profession. Further research is needed to investigate what determines students’ ambition and motivation to study medicine.

We used migratory background as a proxy for the cultural background as it is generally the common way of reporting on ethnicity used in Germany. Although an additional variable on participants’ sense of belonging to a particular culture has been collected for the SEKHHA study, it did not appear to be a reliable variable and therefore it was not used in the stav study, nor reported in the current publication.

Although we report on participants’ gender, the current study is mainly focused on the migratory background and SEB; subsequent studies should investigate the role of gender, particularly in the context of an increasingly female-dominated medical profession.

Finally, there are different dimensions of diversity that deserve researchers’ attention such as, for example, personality traits and risk or time preferences, which can influence treatment choices and adherence [50,51].

## 5. Conclusions

People from lower socio-economic backgrounds and minority groups are underrepresented amongst physicians, medical applicants, and students in Germany. This may be a result of an unjust education system, which mostly favors affluent households and potentially discriminates against migratory backgrounds. The results of the current study could guide the development of widening participation strategies aimed at increasing the diversity of the healthcare force. Such strategies could include actively recruiting and sustainably supporting disadvantaged students to guarantee their admission and success in medical schools.

## Figures and Tables

**Table 1:** Descriptive characteristics of study participants.

Characteristic	SEKHHA Survey	Stav Survey			
	Hamburg Physicians (N = 1457)	Hamburg Medical Applicants (N = 717)	Hamburg Medical Students (N = 195)	Medical Applicants in Germany (N = 2013)	Medical Students in Germany (N = 1256)
Female Gender (N)	58.5% (853)	72.1% (517)	66.7% (130)	74.6% (1502)	73.3% (921)
Mean Age (SD)	48.6 (14.6)	22.9 (2.9)	24.1 (4.5)	22.5 (3.2)	22.4 (3.3)
German citizenship (N)	95.2% (1387)	95.8% (687)	97.4% (190)	96.9% (1950)	97.6% (1226)
German native language (N)	88.4% (1288)	90.4% (648)	88.7% (173)	93.2% (1877)	93.8% (1178)
Highschool GPA * Mean (SD)	1.9 (0.6)	1.7 (0.4)	1.5 (0.5)	1.7 (0.5)	1.6 (0.5)
Parent physician Mean (SD)	21.4 (312)	20.1 (144)	17.4 (34)	18.9 (381)	19.2 (241)
Study financed by parents (N)	69.3% (1010)	78.0% (559)	77.9% (152)	77.0% (1551)	80.4% (1010)
Objective SEB ** Mean (SD)	16.6 (3.4)	17.6 (2.8)	18.0 (2.3)	17.6 (2.7)	17.8 (2.6)
Subjective SEB *** Mean (SD)	6.4 (2.0)	6.6 (1.8)	6.7 (1.8)	6.6 (1.8)	6.8 (1.7)

* Theoretical range: 1.0 (excellent)—4.0 (sufficient); ** Theoretical range: 3–21; *** Theoretical range: 1–10.

**Table 2:** Main cultural groups in our study compared to census data in Hamburg and Germany.

		For Each Cultural Subgroup: N (% of General Population)% of Population with Migratory Background					
	German	Turkish	Polish	Russian	Afghan	Kasach	Syrian
**Germany**							
German census– general population *	59,564,000 (72.7%)	2,747,000 (3.4%) **12.3%**	2,176,000 (2.7%) **9.8%**	1,303,000 (1.6%) **5.8%**	337,000 (0.4%) **1.5%**	1,252,000 (1.5%) **5.6%**	1,052,000 (1.3%) **4.7%**
German census– population aged 20–25 *	2,963,000 (66.3%)	226,000 (5.1%) **15%**	97,000 (2.2%) **6.4%**	61,000 (1.4%) **4.0%**	53,000 (1.2%) **3.5%**	56,000 (1.3%) **3.7%**	105,000 (2.3%) **7%**
Medical applicants in Germany (stav)	1536 (76.3%)	47 (2.3%) **9.9%**	31 (1.5%) **6.5%**	25 (1.2%) **5.2%**	19 (0.9%) **4.0%**	24 (1.2%) **5.0%**	13 (0.6%) **2.7%**
Medical students in Germany (stav)	963 (76.7%)	23 (1.8%) **7.8%**	20 (1.6%) **6.8%**	14 (1.1%) **4.8%**	11 (0.9%) **3.8%**	15 (1.2%) **5.1%**	5 (0.4%) **1.7%**
**Hamburg**							
Hamburg census– general population **	1,189,365 (62.6%)	95,007 (5.0%) **13.4%**	73,444 (3.9%) **10.3%**	36,883 (1.9%) **5.2%**	51,006 (2.7%) **7.2%**	20,676 (1.1%) **2.9%**	20,584 (1.1%) **2.9%**
Hamburg physicians (SEKHHA)	1037 (71.2%)	33 (2.3%) **7.9%**	34 (2.3%) **8.1%**	17 (1.2%) **4.0%**	16 (1.1%) **3.8%**	6 (0.4%) **1.4%**	11 (0.8%) **2.6%**
Medical applicants in Hamburg (stav)	510 (71.1%)	21 (2.9%) **10.1%**	9 (1.3%) **4.3%**	10 (1.4%) **4.8%**	19 (2.6%) **9.2%**	6 (0.8%) **2.9%**	8 (1.1%) **3.9%**
Medical students in Hamburg (stav)	131 (67.2%)	7 (3.6%) **10.9%**	2 (1.0%) **3.1%**	3 (1.5%) **4.7%**	5 (2.6%) **7.8%**	3 (1.5%) **4.7%**	1 (0.5%) **1.6%**

* Statistisches Bundesamt, Bevölkerung mit Migrationshintergrund, Ergebnisse des Mikrozensus 2021; ** Statistisches Amt für Hamburg und Schleswig-Holstein, Bevölkerung mit Migrationshintergrund in den Hamburger Stadtteilen 2021.

**Table 3:** Logistic regression estimating the probability of being admitted into medical school in Germany.

	*p*-Value	OR	95% CI
SEB	<0.001	1.070	1.028–1.103
High school GPA	<0.001	0.370	0.305–0.449
Migratory background	0.217	1.154	0.919–1.450
Gender	0.014	1.320	1.057–1.648

## Data Availability

The data presented in this study is not publicly available and cannot be shared due to privacy and ethical restrictions.
